# Workplace Bullying among Healthcare Workers

**DOI:** 10.3390/ijerph10083121

**Published:** 2013-07-24

**Authors:** Antonio Ariza-Montes, Noel M. Muniz, María José Montero-Simó, Rafael Angel Araque-Padilla

**Affiliations:** Universidad Loyola Andalucía, C/Escritor Castilla Aguayo, 4, Córdoba 14004, Spain; E-Mails: noelmunic@yahoo.com (N.M.M.); jmontero@etea.com (M.J.M.-S.); raraque@etea.com (R.A.A.-P.)

**Keywords:** European Working Conditions Survey—2010, healthcare workers, regression model, working conditions, workplace bullying

## Abstract

This paper aims to assess consistent predictors through the use of a sample that includes different actors from the healthcare work force to identify certain key elements in a set of job-related organizational contexts. The utilized data were obtained from the 5th European Working Conditions Survey, conducted in 2010 by the *European Foundation for the Improvement of Living and Working Conditions*. In light of these objectives, we collected a subsample of 284 health professionals, some of them from the International Standard Classification of Occupations—subgroup 22—(ISCO-08). The results indicated that the chance of a healthcare worker referring to him/herself as bullied increases among those who work on a shift schedule, perform monotonous and rotating tasks, suffer from work stress, enjoy little satisfaction from their working conditions, and do not perceive opportunities for promotions in their organizations. The present work summarizes an array of outcomes and proposes within the usual course of events that workplace bullying could be reduced if job demands were limited and job resources were increased. The implications of these findings could assist human resource managers in facilitating, to some extent, good social relationships among healthcare workers.

## 1. Introduction

Organizations are never neutral; rather, they become a means to crystallize specific socioeconomic interests. In the contexts of maximizing profit and exploiting centrality within work processes, workplace bullying might even be considered an event that can be expected to occur with a certain regularity and frequency. This reality drives researchers to pay special attention to the sources, means, and dynamics generated by power inequality in labor settings [1].

In this line of thought, the phenomenon of workplace bullying has a detrimental effect on both individuals and organizations (e.g., managerial costs and turnover escalate and productivity declines) as the number of witness distractions and the emotional/physical health of the victims increase. Such cases become exponentially worse when a potential lawsuit for unjust dismissal or work compensation/disability is added to an already unfavorable situation. Other economic pitfalls, with a significant negative impact on profits, can sometimes be difficult to measure and clearly define. These pitfalls may include a reduction in the quality, negative impacts on the organization’s reputation, the escalation of mistakes and absenteeism, and the corrosion of customer relationships due to a lack of attention paid to their objectives and commitments, among others [2]. This assertion becomes even more significant for those organizations mainly composed of employees providing particular assistance in a close and direct way to patients (e.g., healthcare workers).

This paper was written in accordance with previous studies that clarify how workplace bullying among healthcare workers has become a persistent phenomenon within organizations. In this sense, Rowell states that, at present, workplace bullying has particularly increased in the health and community care sectors and that such behavior is four times more prevalent in this sector than sexual harassment [3]. In line with these findings, DuHart reports that physicians and nurses are occasionally victims of workplace hostility [4]. The physical violence rates against doctors and nurses are 16.2 per 1,000 and 21.9 per 1,000, respectively. In the European Union, 52% of healthcare jobholders have experienced some sort of aggression at work, followed by 39% of social care workers and 25% of service workers [5].

In the scientific literature, several types of bullying have been studied [6]: intimidation, harassment, victimization, aggression, emotional abuse, and psychological harassment or mistreatment at workplace, among others. The variation in definitions may hinder the conceptualization of the workplace-bullying phenomenon in a more consistent way, inhibiting effective contributions among researchers and practitioners [7]. Bullying is commonly defined by its social manifestations, which are clearly classifiable under the same umbrella as aggressive behavior [8] that generally occurs during interpersonal interactions in work settings [9]. Similarly, there seems to be a consensus that bullying, as a behavior, can be defined in terms of intentionality, frequency (e.g., weekly) or duration (e.g., approximately six months), the targets’ reaction(s), perceived imbalance and misuse of power between the perpetrator and target, inadequate support, and the target’s inability to defend himself from such aggression [10,11,12,13], as well as having to cope with negative and constant social interactions [13], physical or verbal badgering, insulting remarks [12], and intense pressure [14].

Regarding the extent of its manifestation, there is a strong disagreement about the prevalence of bullying; estimates range from 4% to 5% in Northern European Countries [15,16] to 15% in Southern European nations [17]. Certain factors, such as cultural characteristics and social changes, seem to explain the variations in these prevalence rates, as do issues related to research methodology [15]. For instance, studies on workplace bullying have utilized a wide variety of measurement methods, instruments, and research designs [18,19], to the extent that it appears reasonable to consider certain methodological procedures to be biased with respect to their reported prevalence rates [20].

Statistics paint a bleak picture regarding the exposure of healthcare employees to hostility, mostly because bullying at work in the context of healthcare services includes interactions among such varied groups as co-workers, supervisors, patients, families, visitors, and others [21].

Although previous definitions shown a propensity to combine the persistence and duration of the bullying into the same key construct of this phenomenon, the present paper posits that workplace bullying involves a strong psychological component in its materialization. Thus, the main objective of this research was to identify the determinants of workplace bullying among healthcare professionals that emerge from personal variables, working conditions, and contextual factors. In fact, an essential condition of bullying is that the act itself must be perceived as a hostile situation by the target [10,22]. From this point of view, the pernicious effects of workplace bullying (e.g., anxiety, depression, absenteeism, and lack of organizational commitment) are externalized with a greater magnitude at the moment the victim perceives the unpleasant condition, independently of the persistence or duration of the bullying action.

### 1.1. Factors Influencing Workplace Bullying

Given the negative consequences of workplace bullying on the mental health and well-being of employees and, hence, on the performance of any organization, it is vitally important to understand the reasons that trigger the emergence and development of this social phenomenon [23]. In this respect, the psychologists currently leading this specific research approach have mostly focused on victim and/or bullying pathologies.

From a humanistic perspective, this predominantly psychological scope has been utilized to address workplace bullying at an individual level, and many of the studies conducted have been clearly linked to emotional effects and therapeutic practices. This concrete research field has provided a sufficiently broad view and a group of scholars that study the influence of micro-organizational factors (e.g., role conflict, leadership, political aspects, or organizational culture) on individual conduct [12].

Given the above, it appears that research on workplace bullying has evolved towards a multi-causal understanding. In this respect, Hoel and Cooper identified five core areas of focus: subjects, social interaction, group dynamics, working environment, and organizational, societal and political levels [24]. Nevertheless, a considerable number of experts agree that workplace bullying happens as the result of specific interactions among the factors that influence the individual, organizational, or contextual milieu of the people involved [25,26,27,28].

### 1.2. Individual Factors for Workplace Bullying

Some personal characteristics of the victims might constitute potential workplace bullying triggers. In fact, early studies on the subject indicated that employees who experienced conflict at work also experienced similar situations in other contexts, such as with their partners, family, and friends [29]. The perspective of individual antecedents related to workplace bullying has been a controversial topic, as the results are often interpreted as “blaming the victim” [30]. However, studies aiming to identify personality types that are specifically inclined to bullying are far from conclusive [31]; the majority of researchers believe that a personal predisposition for playing the role of victim or bully might not exist [32,33].

Nonetheless, some studies have attempted to identify a selection of individual factors (e.g., gender, age, and seniority) that may increase the risk of becoming a victim or bully [27,32]. The presence or absence of these variables could influence bullying ratios [34], *i.e.*, when bullies weigh the potential costs and personal benefits of their actions based on the particular characteristics of their victims. In this sense, certain groups are considered more vulnerable than others (e.g., women or junior employees).

One of the crucial factors that may be utilized to study bullying on the individual level is gender, although the current results of empirical studies do not quite seem categorical. Some authors have observed a higher frequency of bullying among women compared to men [26,35,36,37,38], while other large-scale studies indicate that, except for sexual harassment, both men and women are equally prone to being bullied at work [19,39,40,41,42].

In any case, Einarsen *et al*. suggest that the gender differences found by some researchers are in fact consequences of the discrimination that both genders can suffer as a result of their position within an organization [12]. From this perspective, one bullying action could perfectly correspond to a concrete behavior oriented toward a specific minority at work, regardless of the gender of this minority. In research concerning nursing staff in Norway, a profession in which men are underrepresented, Eriksen and Einarsen found that female bullying actions reached 4.3%, while this parameter among males escalated to 10.2% [43].

Findings related to a different personal factor, such as the victim’s age, have not identified a clear association with workplace bullying. Referring to this lack of association, Rayner reports that bullying victims are normally under 25 [40]; similarly, Hoel and Cooper find that young people are more likely to experience a greater level of bullying in comparison with older employees [39]. The exact opposite findings are reported by Einarsen *et al*. and Einarsen and Skogstad, who observed a higher incidence of bullying among senior employees [22,44]. This conclusion is also reached by Vartia and Piirainen *et al*. in subsequent research [45,46].

### 1.3. Organizational Factors for Workplace Bullying

The conceptualization of any organization as a whole entity is essential to understand the phenomenon of bullying; it seems quite complicated to imagine a labor context as excessively independent or as non-influential enough to impede internal workplace bullying. Therefore, although early studies have focused mainly on the psychological characteristics of bullies and their victims, several scholars have more seriously pondered the influence of the specific working and structural characteristics of organizations on people. In this paper, we present a brief bibliographical review of the core studies in which the relationships among a number of internal dynamics (e.g., job stability, job design, and human resources practices) and workplace bullying is analyzed.

#### 1.3.1. Job Stability

The level of labor stability might influence the degree of vulnerability to bullying, not only because unstable and temporary jobs are frequently held by lower-status professional employments but also because insecurity reduces the perceived power of employees vis-à-vis their superiors. An empirical exploration among university employees at a specific academic center was conducted in a noteworthy effort to demonstrate that flexible working arrangements can contribute to the prevalence of bullying [47]. In fact, one of the reasons given to explain the increase of bullying within 21st century organizations is that the organizational restructuring processes and higher levels of outsourcing have enlarged the power gap between managers and employees [48,49].

In this scenario, we could take for granted that the bullying rates among employees with temporary contracts would be higher than rates registered among their colleagues with permanent contracts. However, Kivimäki, Elovainio, and Vahtera do not observe any difference between these two groups, or between full-time and part-time employees [50]. In reference to these differences, the research results seem to be conflicting as well; while Baron and Neumann find a positive relationship between part-time employment and bullying, Hoel and Cooper report the same finding, but among full-time employees [39,51].

#### 1.3.2. Intrinsic Characteristics of Job Position

The empirical research on workplace bullying and the intrinsic characteristics of job position is also extensive. Previous studies have identified certain variables, such as workload [26,39,44], control [36,41,52,53,54], role ambiguity [44,55], role conflict [44,56], leadership behavior [41,44], social support from co-workers and supervisors [36,57], social climate [36,39,41,44,53,58], and organizational change [39,59,60,61], as the key elements predicting the occurrence of bullying within organizations.

A large investigation conducted in the United Kingdom on 5,200 subjects reveals that victims of workplace bullying, compared to non-bullied individuals, are distressed by their workload, rarefied working environment, greater organizational change, unsatisfactory relationships at work, and a more consistent intention to resign [39]. Similarly, a study on Norwegian employees by Einarsen *et al*. reports a significant correlation between the variables described above and workplace bullying (*i.e.*, workload, control, role ambiguity, role conflict, leadership behavior, social climate, and organizational change) [44]. Similarly, Salin finds that bullying appears to be correlated with politicized and competitive organizational climates and even slightly more strongly with workload [26]. Correspondingly, in a sample of 400 employees from five Swedish organizations, Hansen *et al*. observe a negative correlation between bullying and the support given to employees by their colleagues and their superiors [57].

Bowling and Beehr’s meta-analysis, which reviewed over 90 studies published between 1987 and 2005, makes a remarkable contribution to the investigation of workplace bullying by compiling and organizing the extant empirical research [31]. Regarding the characteristics of a job position, these authors report that bullying tends to emerge in occupational settings where other stressors, such as role conflict (r = 0.44), role ambiguity (r = 0.30), overload (r = 0.28), and work limitations (r = 0.53), are often simultaneously identified. Likewise, they confirm that autonomy at work is negatively associated with bullying (r = −0.25).

Further organizational variables that have been studied for their associations with bullying include monotony, complexity, and teamwork. Zapf *et al*.’s research makes evident that monotonous and repetitive tasks are more frequent among bullying victims [36]. Correspondingly, in a subsequent investigation, Zapf does not corroborate any association between bullying and work complexity [28]. Similarly, Zapf *et al*. realize that, during activities requiring teamwork, bullying among peers seems to be more likely to occur [36]. According to those authors, the social environment generated within these groups contributes to the search and selection of scapegoats among the less powerful members to redirect team aggressiveness.

Moreover, numerous analyses have validated the connections between workplace bullying and individual perceptions within organizations, such as job satisfaction and commitment. The former has been amply studied by Vartia and Hyyti and constitutes an additional and plausible alternative variable related to bullying [53]. Job dissatisfaction, which causes victims emotional distress, can be considered a condition necessarily linked to affective commitment. However, quite a few authors have reported a negative relationship between these two variables [39,62]. Employees who are highly committed to their organizations may be more vulnerable to stressors in their working environment precisely due to their emotional ties their social structures [63].

#### 1.3.3. Occupation and Bullying

The academic literature is expanding with a prolific number of studies concerning bullying in specific types of occupations. On this subject, certain authors report that 44.0% of nursing staff members have been bullied at some point in their working lives [64]. Other occupations with high incidences of bullying include restaurant employees [65], teachers [66], university professionals [34,67,68], business professionals [69], transportation workers [68], and police officers [70]. Diverse investigations have identified multiple occupations within the same studies: blue-collar workers, clerks and service workers, associate professionals, managers and professionals, among others [71,72].

Related to this issue, Woodman and Cook report interesting results in the UK utilizing a sample of 512 managers; 39.0% of the respondents affirmed that they had been bullied in the past three years [73]. Bullying appears to have detrimental effects at all management levels; middle managers, as an example, appear only slightly more prone to suffer workplace bullying, representing 49.0% of the reported cases during the past three years. This figure may support the phenomenon known as “*management squeeze*”, in which middle managers are subjected to the particular pressures of being required to implement unpopular policies as a result of the decisions made at more senior levels. In previous studies, Ariza, Morales, and Menor identify assorted factors that may contribute to the emergence of workplace bullying within managers [74]. Apparently, the likelihood of a manager being bullied increases when job insecurity is present, when people are dissatisfied with their work and salary, when employees are predominantly in the public sector, and when work activities are very emotionally demanding.

### 1.4. Contextual Factors in Workplace Bullying

In addition to the factors related to the internal dynamics of organizations, bullying may also be occur as a result of the context in which the organization operates. Research on this subject reveals that bullying is more frequent in the service sector than in any other industry, particularly in health, public service, education, and financial service [34,52]. Furthermore, Leymann argues that bullying most commonly occurs in the health care sector, especially among nurses, due to their work overload and the double supervision they are subjected to by doctors and chief nurses, which violates the Unity of Command Principle [42]. Supporting this argument, Yildrim and Yildrim affirm that 87.0% of nurses in Turkey have experienced some form of bullying, especially those in the public sector [75].

High levels of bureaucracy, the existence of very strict norms, and excessively high job security may generate environments amenable to the occurrence of bullying, as these settings make bullies invisible and victims less likely to resign [69]. In this sense, Zapf *et al*. provide a wide-ranging summary of European studies and conclude that the prevalence of bullying is higher for the public sector, service, health, education, and assistance fields than for private industry [19]. A similar conclusion is presented by Giorgi, Arenas, and Leon-Perez in Italy [17] and by Hoel and Cooper in the United Kingdom [39]. These authors report more significant bullying activity within public services (e.g., education or correctional assistances) and a lower prevalence of bullying in the retail and industrial sectors. Similarly, Soares’ research shows that 4.4% of public education and health care employees have been bullied occasionally by their patients or students while completing their daily tasks [76].

Furthermore, LaVan, Katz, and Jedel suggest that public sector jobholders should manage their employment relationships differently than do workers in the private sector. This difference may lead to an alternative form of workplace bullying [77]. Although there are some research papers suggesting that bullying might be higher within the public sector [19,39,69,78], LaVan, Katz, and Jedel firmly believe that this actually occurs because countless jobs in the public sector entail a great deal of emotional labor rather than instrumental work [19,77,79]. Public sector employees enjoy a special type of employment status; they are protected by civil service rules and regulations, by unions with internal grievance procedures, and by statutes that provide protection against retaliation for whistle blowing.

For the purpose of the present work, a comprehensive workplace-bullying model is proposed in the following section. Then, the most relevant empirical results obtained through a logistic regression analysis are presented, followed by the main conclusions and limitations of the study.

## 2. Methods

The data utilized for this research were obtained from the 5th European Working Conditions Survey, conducted in 2010 by the *European Foundation for the Improvement of Living and Working Conditions*. This survey provides insight into to the working environment and employment conditions of the 27 EU Member States, including Albania, Croatia, Kosovo, Macedonia, Montenegro, Norway, and Turkey. The target population includes workers aged 15 years and over (16 and over in the case of Spain, the UK, and Norway) who are employed and reside in the country being surveyed. This was a multi-stage investigation using a stratified random sample. Over 43,000 interviews were collected in 2010. The study found that the prevalence rate of workplace bullying was 11.3% among healthcare workers. Given the objective of this research, we gathered a sub-sample of 284 health professionals, including members of the International Standard Classification of Occupations (ISCO-08) subgroup 22 (e.g., medical doctors, nursing and midwifery professionals, traditional and complementary medicine professionals, dentists, ophthalmic opticians, and physiotherapists). In total, 41.2% of these health professionals claim to have experienced workplace bullying (N = 117), while 58.8% indicate that they have not (N = 167).

The subjects in this sample are medical doctors (66.9%), nursing and midwifery professionals (21.5%), or other health professionals (11.6%). They are drawn from both the public (67.6%) and private sectors (32.4%), are 60.9% female and 39.1% male, and have an average age of 44.1 years. Finally, 11.3% completed secondary education, and 88.7% completed their university studies. The term “secondary education” is used to categorize individuals with either high school study or vocational/technical training.

The dependent variable for this analysis is bullying at work. Respondents were asked to answer just one question based on their individual experience: *Over the past 12 months, during the course of your work, have you been subjected to bullying/harassment?*

Bullied professionals are codified as 1, while those who claim not to have felt bullied are coded as 0. Two main approaches are used in the bullying research questionnaires were implemented: the self-labeling and operational approaches. The limitations and advantages of these methods are discussed in Nielsen *et al*. [80].

Workplace bullying is considered in this study to be a complex phenomenon that arises due to the dynamic interactions of labor environment variables and individual factors. Taking into account preceding studies on bullying at work, this study is arranged in three sets of independent variables grouped into three categories: personal and family factors, working conditions factors, and organizational/contextual factors. The codes and classification of explanatory variables are as follows: 

*Individual characteristics*: *Gender* (0: Male; 1: Female), *Age* (0: 15–24; 1: 25–39; 2: 40–54; 3: 55 or over), *Level of education* (0: University education, 1: Secondary education), *Marital status* (0: Partnered; 1: Single), and with *Children at home* (0: Yes; 1: No).

*Working conditions*: *Length of service* (0: more than 10 years; 1: more than 5 up to 10; 2: more than 1 up to 5; 3: up to one year), *Type of contract* (0: A permanent contract; 1: A temporary contract), *Working hours* (0: More than 40 h; 1: 20 to 40 h; 2: Less than 20 h), *Work at night* (0: No; 1: Yes), *Work on Sundays* (0: No; 1: Yes), *Working day* (0: Full time; 1: Part time), *Shift work* (0: No; 1: Yes), *Monotonous tasks* (0: No; 1: Yes), *Complex tasks* (0: Yes; 1: No), *Rotating tasks* (0: No; 1: Yes), *Team work* (0: No; 1: Yes), *Flexibility in work methods* (0: Yes; 1: No), *Work stress* (0: No; 1: Yes), *Working conditions satisfaction* (0: Yes; 1: No), *Pay satisfaction* (0: Yes; 1: No), *Likely to be dismissed* (0: No; 1: Yes), *Expectation of career growth* (0: Yes; 1: No) and *Motivation* (0: Yes; 1: No).

*Organizational context*: *Type of sector* (0: Private; 1: Public) and *Size* (0: Micro enterprise (1–9 employees); 1: Small enterprise (10–49 employees); 2: Medium-large enterprise (50+ employees)).

The IBM SPSS 20 (Statistical Package for Social Science) software application was utilized to measure the variables. The methodology employed to accomplish the objectives was based on the binary logistic regression model, a specific type of regression model intended for dichotomous variables. This statistical technique is used to determine the probability that an event will happen (workplace bullying, in this case) compared to the probability that it will not.

## 3. Results

Table 1 lists some of the main sociodemographic characteristics of both the healthcare workers who reported that they were bullied (N = 117) and those who did not (N = 167).

**Table 1:** Distribution of both bullied and non-bullied healthcare workers in their labor environments according to sociodemographic and working characteristics.

Characteristic	Sociodemographic and work-related factors among health care workers who were bullied in their workplaces (N = 117)		Sociodemographic and work-related factors among health care workers who were bullied in their workplaces (N = 167)	
	Number (n)	Percentage (%)	Number (n)	Percentage (%)
**Gender**				
Male	32	27.4%	79	47.3%
Female	85	72.6%	88	52.7%
**Age**				
15–24 years	2	1.7%	2	1.2%
25–39 years	54	46.2%	45	27.3%
40–54 years	50	42.7%	75	45.5%
55 years or older	11	9.4%	43	26.1%
**Level of education**				
Secondary education	25	21.4%	7	4.2%
University education	92	78.6%	160	95.8%
**Employment contract**				
Long-term contract	91	85.0%	83	78.8%
Temporary contract	16	15.0%	28	25.2%
**Management position**				
Yes	18	15.8%	54	33.1%
No	96	84.2%	109	66.9%
**Sector**				
Public	78	66.7%	93	55,7%
Private	22	18.8%	60	35.9%
Other	17	14.5%	14	8.4%
**Job satisfaction**				
Yes	78	67.2%	146	88.0%
No	38	32.8%	20	12.0%
**Stress**				
Yes	105	89.7%	129	77.2%
No	12	10.3%	38	22.8%

Furthermore, some statistical differences were observed regarding the array of variables. Workplace bullying emerges as even more acute among female healthcare workers (72.6% compared to 52.7%; χ^2^ = 11.507, d.f. = 1, *p* = 0.001) young workers (46.2% compared to 27.3% between 25–39 years old; χ^2^ = 17.107, d.f. = 3, *p* = 0.001), workers who did not attend university (21.4% compared to 4.2%; χ^2^ = 20.301, d.f. = 1, *p* = 0.000), workers with a permanent contract (85.0% compared to 78.8%; χ^2^ = 3.568, d.f. = 1, *p* = 0.042), workers not in management positions (84.2% compared to 66.9%; χ^2^ = 10.485, d.f. = 1, *p* = 0.001), workers in the public sector (66.7% compared to 55.7%; χ^2^ = 8.182, d.f. = 1, *p* = 0.004), workers who are not satisfied with their jobs (32.8% compared to 12.0%; χ^2^ = 17.927, d.f. = 1, *p* = 0.000), and workers who experience stress in their daily work (89.7% compared to 77.2%; χ^2^ = 7.408, d.f. = 1, *p* = 0.004), compared to healthcare professionals who do not consider themselves as bullied in their workplaces.

Table 2 shows the results for the estimations calculated by logistic regression that were derived from the factors determining the level of workplace bullying within health professional settings. The statistical tests applied to assess the validity of the model (Hosmer-Lemeshow analysis; Chi-square test: 5.444; Sig. 0.709) largely suggested enough basis to acknowledge its validity; that is, they affirmed that the set of job-related variables considered for the general model of this research may potentially explain in a satisfactory manner whether a health professional is prone to experiencing bullying at work. We should also highlight that the chosen variables allow the model to be generalized, indicating its possible utility for predictive purposes. The logistic regression model integrates individual and organizational factors and estimating that the likelihood of workplace bullying is 80.8% (87.6% for bullied healthcare workers and 71.6% for non-bullied ones).

**Table 2:** Logistic regression for factors that may determine workplace bullying (confidence intervals for odds ratios).

Variables in the model	B	S.D.	Wald	Sig.	Odds ratios 95% C.I. for OR		
					OR	Lower	Upper
Gender (0: Male; 1: Female)	1.020	0.364	7.829	0.005	2.772	1.357	5.662
Age (0: 15–24; 1: 25–39; 2: 40–54; 3: 55 or over)	−0.466	0.253	3.393	0.065	0.627	0.382	1.030
Level of education (0: University education, 1: Secondary education)	1.706	0.574	8.843	0.003	5.507	1.789	16.951
Children at home (0: Yes; 1: No)	1.053	0.428	6.058	0.014	2.867	1.239	6.632
Shift work (0: No; 1: Yes)	0.986	0.348	8.024	0.005	2.682	1.355	5.307
Monotonous tasks (0: No; 1: Yes)	0.790	0.353	5.009	0.025	2.202	1.103	4.397
Rotating tasks (0: No; 1: Yes)	0.956	0.371	6.627	0.010	2.602	1.256	5.388
Work stress (0: No; 1: Yes)	1.602	0.546	8.622	0.003	4.962	1.703	14.456
Working condition satisfaction (0: Yes; 1: No)	0.889	0.417	4.554	0.033	2.434	1.075	5.509
Expectation of career growth (0: Yes; 1: No)	1.508	0.393	14.691	0.000	4.517	2.089	9.765
Constant	−4.408	1.020	18.683	0.000	0.012		

In general, the model brings to light the extent to which the probability of a health sector employee considering him/herself bullied is higher among younger and less educated women who have small children at home, are unsatisfied with their working conditions (e.g., working in shifts or performing monotonous and rotational assignments), and suffer from work stress and lack of promotion opportunities within their organizations.

As a consequence, the personal and organizational variables appeared reliable for predicting the development of certain attitudes, such as workplace bullying, and for identifying critical constructs for the understanding of the phenomenon under study. Each of the personal and job-related significant factors that measure the probability of workplace bullying have a different degree of impact, as indicated by the analysis of the confidence intervals obtained in the corresponding *odds ratios* (see Table 2). Accordingly, the most influential variables related to workplace bullying are the level of education (OR 5.507; CI 1.789-16.951), work stress (OR 4.962; CI 1.703-14.456), and expectation of career growth (OR 4.517; CI 2.089-9.765). These factors could prove to be strong predictors of whether an employee is exposed to bullying or not.

The odds ratio coefficients for other variables (gender, children at home, shift work, monotonous tasks, rotating tasks, and working condition dissatisfaction) remain over 2.0, with the exception of age, with an odds ratio coefficient of 0.627 and a confidence interval ranging from 0.382 to 1.030.

## 4. Discussion

Advances in understanding the primary circumstances that precede workplace bullying take on great importance in the development of more effective prevention and intervention tools to remedy this social problem [81,82]. Workplace violence in healthcare settings occurs four times more often than in all other private-sector industries combined [83], with the highest incidences in psychiatric wards, accident and emergency departments, and high-dependency units [84]. The bullying prevalence varies significantly from one country to another and even within the same country. In Europe, for example, even though the inferences may vary depending on the measurement and estimation methods being utilized [15,85], studies of the occurrence of workplace bullying report rates of approximately 4–10% [19]. For the present research, the prevalence rate happens to be slightly higher: 11.3% of healthcare workers labeled themselves as bullied in their professions. Nevertheless, this ratio is still far from the results obtained in the U.S., where 38% of the healthcare employees report psychological harassment [86]. This relationship is similar to that described by Dellasega, who finds that 44.0% of nurses experience episodes of bullying at some point during their working lives. [64].

These results indicate that the rate of workplace bullying for health professionals is larger than the predicted average calculated from similar parameters for employees laboring in any other occupational sector. Regarding this point, Zapf *et al*. provide an extensive summary of European studies and conclude that the prevalence of bullying is higher in the public sector (e.g., service, health, education, and assistance) than in the private sector [19]. A similar conclusion is reached by Hoel and Cooper in the United Kingdom, who report a higher incidence of bullying within public services, such as education or correctional assistance, and a lower incidence in the retail and industrial sectors [39]. Similarly, Soares’ research shows that 4.4% of public education and health care employees have been occasionally bullied by their patients or students while completing their daily tasks [76].

A body of literature has emerged describing the possible triggers of workplace bullying within healthcare staff and has focused primarily on two areas. The first area pertains to the personal and individual differences among those involved in the bullying incidents, while the second pertains to the characteristics of the surrounding organizational settings in which these circumstances occur. Similar to the present paper, many authors currently embrace the concept that both individual and organizational factors are important to understanding bullying behaviors. For instance, healthcare workers under 40 are the most frequent victims of violent events [87]. Researchers have also observed that older workers experience significantly less violence than young workers [88,89,90,91,92]. Other characteristics of healthcare workers that have been associated with an increased risk of workplace bullying include gender and marital status. Furthermore, a greater percentage of female physicians fear a potentially violent encounter at work compared to male physicians [93]. Lin and Liu’s study reports that unmarried workers are significantly more likely to experience workplace violence compared to married employees [94]. In the European Union, these results suggest that there are specific sociodemographic features that may influence the phenomenon of workplace bullying. Regarding this concern, the current regression analysis outcomes indicate a tendency for young female healthcare workers with only secondary education and with children at home to suffer bullying. The group described above is certainly in a position of greater weakness in relation to the other groups that have greater power, for example, men with university degrees or higher seniority in their organizations. Additionally, regarding concrete cases related to female jobholders, persistent and predominant sexist attitudes should be noted, as well as structural barriers that inhibit women’s careers to a certain extent compared to their male colleagues. These circumstances make these groups particularly more vulnerable; therefore, they are more likely to end up as victims of workplace bullying.

Furthermore, some organizational factors are found to increase the odds of workplace bullying against healthcare workers. For instance, with respect to working conditions, McAneney and Shaw report that violent events in long-term care are more likely to take place during the evening and night hours [95]. It has been claimed in recent meta-analyses that there are some specific organizational variables (e.g., workplace bullying antecedents) that are worth noting, such as conflict and role ambiguity [31], work overload, stress, lack of autonomy, and absence of organizational fairness [56]. Zapf *et al*.’s research shows that performing monotonous and repetitive tasks is more common among bullying victims [36]. At the organizational level, this study emphasizes that the propensity for a healthcare worker to experience bullying escalates among those who work on a shift schedule, perform monotonous and rotating tasks, suffer from work stress, experience a lack of satisfaction due to their working conditions, and do not perceive any opportunity for promotion within their organization. This unpredictable environment, characterized by insecurity, role conflict, and tension, allows few opportunities for socialization and even less time for conflict resolution; both of these factors may indirectly contribute to the emergence of aggressive behaviors and bullying. Ultimately, a stressful social climate and precarious work atmosphere create a breeding ground for workplace bullying, as reflected in the results of the present empirical study.

Finally, it seems accurate to say that the contextual variables of an organization do not influence workplace bullying. Given this finding, it is possible to deduce that workplace bullying is prevalent with the same degree of intensity in both public and private organizations, regardless of their size.

## 5. Conclusions

Workplace bullying has become a serious and growing problem that affects a significant proportion of healthcare professionals. As a result of its negative consequences on the mental health and well-being of employees, and hence on the performance of the organizations, the importance of understanding the factors that contribute to the emergence and development of bullying is vital [23]. In this regard, the present study aims to contribute to the development and implementation of measures to prevent bullying in the health sector.

The multidimensional model created in the present research is intended to identify healthcare workers who are prone to being bullied at work as individuals; the study findings have valuable and pertinent implications for institutions that aim to thrive and to enhance organizational performance. This work provides reasonable evidence that could be of significant benefit in the implementation of human resource policies: responsible managers could reduce the organization-wide levels of workplace bullying by adjusting certain working conditions that negatively affect employees who are especially susceptible to being bullied, given their personal characteristics.

This research paper offers an empirical basis for further studies related to health sector issues in Europe. Attracting and retaining the most qualified and experienced professionals has become essential for successful and competitive organizations in the healthcare industry; organizations are urged to implement strategies oriented toward reducing workplace bullying. Consequently, the labor force in this economic sector has specific traits that should not be ignored.

From a practical standpoint, the present findings could assist practitioners in facilitating harmonious social relationships among healthcare workers. Particularly, the results suggest that limiting job demands and increasing job resources could reduce workplace bullying. Specific attention should be paid to young women who feel dissatisfied with their working conditions, as they constitute a group with an increased risk of experiencing bullying.

Despite the significant findings of this study, its intrinsic methodological limitations must be considered. First, the phenomenon of bullying was measured by self-report, which might increase the risk of common method variance, forcing us to assume a corresponding bias in the key variables. Exploring the experiences of 385 self-identified Canadian nurses, we showed that when targets identify themselves as victims, they report decreased job satisfaction, escalation of their level of burnout, and greater psychological distress in comparison with those exposed to bullying but who do not label themselves as sufferers [96]. Second, by utilizing self-identification without a strict definition, bullying is measured in broad terms, and consequently, there is a risk of overestimating its prevalence, as the respondents could report incidents that would not qualify as bullying according to the researchers’ understanding of the phenomenon [20]. Third, a related methodological problem could be social desirability; previous scholars have analyzed the repercussions of desirability in workplace bullying studies. Given the particular understanding of the phenomenon under investigation, it seems probable that any given prevalence rate would exceed the rates obtained in this type of research, as many of the present victims took a large amount of time to acknowledge and accept that they were subjected to aggression of this nature. This predicament is particularly acute among certain population groups that are considered more vulnerable, such as women, young people, and employees with temporary interrelations. To correct this problem, some authors propose to make use of multi-method data and utilize objective measures that may reinforce workplace-bullying research. Examples of this type of data include managerial reports and scores from third parties (e.g., researchers) [97]. It should be noted, however, that assessing third party scores of workplace bullying without trying to counteract these behaviors raises ethical concerns due to the many negative consequences of workplace bullying for the parties involved, as well as for the work unit and the organization [98]. Fourth, the observed correlations between bullying and the variables analyzed in this study should be assessed cautiously, as the data are cross-sectional and not experimental. Finally, this study represents only a partial perspective of this phenomenon: the point of view of the victim but not of the bully.
